# Wide-Field Multi-Parameter FLIM: Long-Term Minimal Invasive Observation of Proteins in Living Cells

**DOI:** 10.1371/journal.pone.0015820

**Published:** 2011-02-02

**Authors:** Marco Vitali, Fernando Picazo, Yury Prokazov, Alessandro Duci, Evgeny Turbin, Christian Götze, Juan Llopis, Roland Hartig, Antonie J. W. G. Visser, Werner Zuschratter

**Affiliations:** 1 Leibniz Institute for Neurobiology, Magdeburg, Germany; 2 University of Castilla-La Mancha, Albacete, Spain; 3 Arivis Multiple Image Tools GmbH, Rostock, Germany; 4 Russian Research Center Kurchatov Institute, Moscow, Russia; 5 Otto-von-Guericke University, Magdeburg, Germany; 6 Wageningen University, Wageningen, The Netherlands; Dalhousie University, Canada

## Abstract

Time-domain Fluorescence Lifetime Imaging Microscopy (FLIM) is a remarkable tool to monitor the dynamics of fluorophore-tagged protein domains inside living cells. We propose a Wide-Field Multi-Parameter FLIM method (WFMP-FLIM) aimed to monitor continuously living cells under minimum light intensity at a given illumination energy dose. A powerful data analysis technique applied to the WFMP-FLIM data sets allows to optimize the estimation accuracy of physical parameters at very low fluorescence signal levels approaching the lower bound theoretical limit. We demonstrate the efficiency of WFMP-FLIM by presenting two independent and relevant long-term experiments in cell biology: 1) FRET analysis of simultaneously recorded donor and acceptor fluorescence in living HeLa cells and 2) tracking of mitochondrial transport combined with fluorescence lifetime analysis in neuronal processes.

## Introduction

Time-domain Fluorescence Lifetime Imaging Microscopy (FLIM) is a groundbreaking tool to study protein interactions in living cells [1]–[3]. In each pixel of time-domain FLIM images, a fluorescence decay signal is recorded by e.g. the Time-Correlated Single Photon Counting technique (TCSPC) [4], [5]. Dissimilarly to CCD imaging, FLIM allows a direct measurement of the fluorescence decay rate of the observed fluorophores and their environment interactions on picosecond/nanosecond time-scale at sub-micrometer space resolution, thereby considerably increasing the information content of the experiment.

Many processes influence the fluorescence decay kinetics of fluorophores in living cells e.g. Förster Resonance Energy Transfer (FRET), excited-state quenching processes, pH concentration as well as the local refractive index and changes of the cells' auto-fluorescence during the exposure time [6]–[8]. Endogenous (chlorophylls, NAD(P)H, flavins) or transfected fluorescent chimeric proteins and biochemical sensors can be imaged to provide insights in the local protein environment. The FLIM technique has been further refined to detect simultaneously multiple physical parameters of the fluorescence radiation in recent years. The detected signal is split into several spectral bands and/or polarization directions, which are simultaneously recorded. However, the illumination light dose is a well known limiting factor for continuous long-term observation of living cells due to photobleaching of the fluorophores and the production of Reactive Oxygen Species (ROS) [9]. In addition, the effects of the excitation intensity levels at a given illumination energy dose on the sample show variable results, which strongly depend on the biological system under investigation [9].

Recently, Controlled Light-Exposure Microscopy (CLEM) has been introduced to minimize the excitation energy dose on the sample in laser-scanning confocal microscopy [10]. The method's strategy is based on the modulation of the excitation energy dose over the field of view depending on the local sample brightness. CLEM measurements succeeded in observing sensitive biological samples continuously over many hours [11]. However, this powerful technique has not been applied to FLIM yet.

In the current work, we present a novel approach to perform multi-parameter FLIM in long-term observations of living cells: Wide-Field Multi-Parameter FLIM (WFMP-FLIM). The WFMP-FLIM setup is based on a conventional fluorescence microscope and a space sensitive photon-counter, the Quadrant Anode photomultiplier (QA) [12]. In combination with wide-field Pulsed Interleaved Excitation (PIE) [13], the WFMP-FLIM setup provides simultaneous two-wavelength illumination of the entire field of view at minimal invasive intensities (
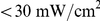
). The use of a Quadrant Anode as a FLIM detector enables the registration of fluorescence decay signals at a very high time resolution down to few picoseconds per time channel. The system is capable to measure directly the transfer rate of fast photochemical processes as, for instance, FRET and to resolve complex fluorescence decay kinetics [14]. However, the limited number of photons available in each single pixel at low excitation intensities (usually 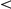
1000) previously restricted the data analysis to the estimation of the average decay time with sufficient accuracy [15]. This limitation is overcome if all the pixels of the available FLIM images are simultaneously analyzed under the hypothesis of global analysis i.e. the spatial invariance of the lifetimes of individual fluorescent species is assumed [16]. The estimation accuracy is further increased by applying the Maximum Likelihood Estimation (MLE) in spite of the Least Square (LS) method [17]. Experiments using a calibration dye solution demonstrate that the WFMP-FLIM setup achieves the highest possible accuracy in the estimation of the model parameters predicted by the MLE theory [18].

WFMP-FLIM offers a broad range of possible applications in long-term live cell imaging. Furthermore, any fluorescence microscope equipped with pulsed-laser illumination sources can be upgraded into a multi-parameter FLIM system by adding a position sensitive photon-counter in combination with a laser synchronization unit.

## Results

### Performances of the data analysis algorithm

The used data analysis technique is a blend of well established methods, i.e. MLE and LS (see materials and methods section).

The algorithm was extensively tested on simulated data-sets. The fit, mainly performed on bi- and tri-exponential fluorescence decay models at variable number of photons, lifetimes and amplitudes, were always convergent to the true set of parameters. Accuracy tests of the MLE and LS methods on synthetic data sets were already published e.g. [17], [19]. Therefore, only the performances on real measurements by the WFMP-FLIM setup are presented in the frame of the current work.

FLIM images of a Rhodamine 6G solution in n-butanol were acquired. The dye decayed with a mono-exponential kinetic when the fluorescence emission was monitored through a polarizer oriented at 54.7 (or magic angle) to the electric field of the laser excitation [6]. When the polarization filter was oriented parallel with the electric field, the dye exhibited a bi-exponential decay kinetic with a fast component of 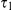  = 0.5 ns (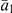 = 10%) and a slow component of 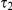 = 3.7 ns (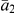 = 90%). These components were attributed to fast de-polarization of the dye and its lifetime in n-butanol respectively. The fast component was originated from the rotation of the emission dipole moment of the molecules in the excited state due to the Brownian motion.

The choice of n-butanol as a solvent was motivated by the obtained fast component of 0.5 ns. In fact, it approximated the quenched decay time of the eGFP in presence of FRET as presented in the following sections, providing a suitable calibration method for the FLIM setup.

**Figure 1:**
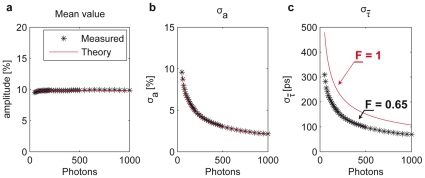
Test of the data-analysis algorithm on real FLIM data. a) Mean and b) standard deviation values of the normalized amplitude of the fast component in the fluorescence decay of a Rhodamine 6G n-butanol solution as a function of the number of photons per pixel. The measured values are shown in black, the values expected by an unbiased estimator which reaches the theoretical limit of the CRLB are given in red. c) Measured standard deviation of the average lifetime 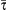 (black) and expected one by a mono-exponential fit assuming a F-value equal to 1 (red).

The data sets were analyzed by selecting variable number of photons per pixel (from 20 to 1000) in order to estimate mean and standard deviations of the pre-exponential factors and average lifetimes as a function of the number of photons. The precision in the determination of the two fluorescence lifetimes in the FLIM image was negligible due to the total number of photons used (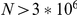). The values obtained are shown in Fig. 1: the mean pre-exponential factors over the pixels were independent from the number of photons used in the analysis for reasonable numbers of photons per pixel (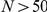). A bias of some percentage was observed for 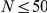. Hence, the used data analysis method provided an unbiased estimation of the model parameters on the analyzed experimental data. The dispersion of the measured amplitudes around the mean value in each pixel (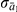) was also calculated. The maximum likelihood theory provides a lower bound in the estimation accuracy of a model parameter (in the current experiment 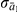): the Cramér-Rao Lower Boundary condition (CRLB) [18]. The CRLB was numerically calculated and plotted as a function of the number of photons per pixels (Fig. 1b). The measured standard deviations (black) and the theoretical ones expected by the CRLB (red) showed very close values for the given model. The presented data analysis method achieved the theoretical limit of the parameter estimation on data acquired by the WFMP-FLIM setup. A maximum deviation of less than 1% from the CRLB limit was observed. The analysis proves that the theoretical limit in fitting bi-exponential models on fluorescence decays with low number of photons is practically achieved on real experimental data.

Another important aspect of the data analysis method implemented for the WFMP-FLIM setup concerns the accuracy in the estimation of average lifetimes (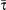, eq. 9) in each pixel. The average lifetimes can be estimated either independently in each pixel of the FLIM image or under the global analysis hypothesis. In the former case, a lower bound in the estimation accuracy of 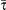 exists: 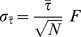
(1)where 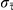 is the standard deviation of the measured 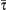 and 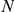 is the number of photons in the pixel. 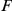 is the *photon economy*, an indicator of the estimation accuracy of a detection system to obtain average lifetimes [20]. The *photon economy* has a lower bound *F*-value equal to one, which is only achieved in an experimental setup whose signal to noise ratio is only limited by the statistical noise. The following equation is valid in this limit case: 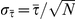. In figure 1c, the measured accuracy in the estimation of 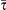 in single pixels under the global analysis approximation (black) is compared with the accuracy expected for an ideal detection system (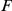 = 1). The former approximation is definitely more accurate in estimating average lifetimes than the independent 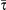 calculation in each pixel.

### Lifetime analysis of long-term experiments on HeLa cells

To demonstrate that the WFMP-FLIM technique can monitor living cells during long periods of continuous observation (up to 10 h) and simultaneously retrieve complex fluorescence decay dynamics, HeLa cells transfected with a tagRFP-5AA-eGFP chimeric protein were measured. The construct consisted of a binary composition of one eGFP and one tagRFP molecule linked by a flexible chain of 5 amino acids. A strong FRET signal was obtained due to the favorable distance between the donor (eGFP) and acceptor (tagRFP) molecules.

**Figure 2:**
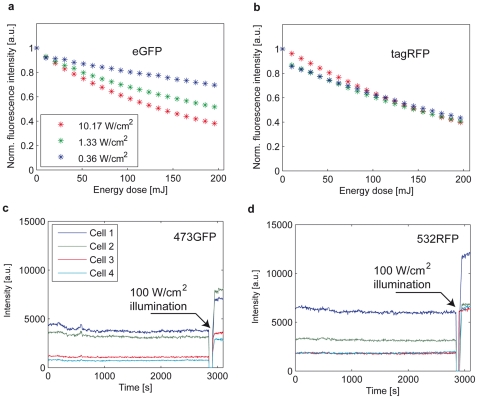
Non-linear photobleaching and ROS production. Dependence of the eGFP (a) and tagRFP (b) fluorescence emission signal as a function of the energy dose at several excitation intensities. In case of the eGFP molecules, the higher the intensity, the faster was the bleaching of the signal in fixed HeLa cells. (c) Long-term illumination of living HeLa cells loaded with 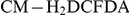 to monitor the production of ROS species in the 473GFP and (d) 532RFP channels. No increase of the fluorescence due to the illumination-induced oxidation of the 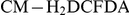 molecules was observed during 45 minutes. Few seconds of strong illumination by a Hg-lamp (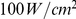 for 5 seconds) were sufficient to generate ROS and increase the fluorescence signal. The intensity of the fluorescence signals is given in arbitrary units [a.u.].

Firstly, the photobleaching of the tagRFP-5AA-eGFP construct at several illumination intensities was investigated. Figure 2a clearly demonstrates that, at a given energy dose, lower illumination intensities (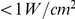, FITC filter set) induced less photobleaching of the eGFP fluorophores. Interestingly the tagRFP fluorophores showed an almost independent bleaching trace as a function of the energy dose used to illuminate the probe (Fig. 2b, TRITC filter set). The low-intensity excitation maximized the number of fluorescence photons which could be emitted by the tagRFP-5AA-eGFP construct before photodamage occurs. Afterwards, long-term experiments on living cells expressing the same construct were performed. Irreversible photobleaching of eGFP and tagRFP was not detectable during many hours of experimental acquisitions.

The photo-production of ROS at typical excitation intensities required by the WFMP-FLIM setup was also monitored. HeLa cells expressing the tagRFP-5AA-eGFP construct were additionally loaded with a ROS sensing dye *(5-(and-6)-chloromethyl-2',7'-dichlorodihydrofluorescein diacetate, acetyl ester* or 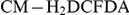), as described elsewhere [21]. During 45 minutes of continuous exposure, no increase of the fluorescence signal due to oxidation of 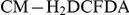 was observed. Conversely, only 5 seconds of epi-illumination by a Hg-lamp (TRITC filter set, 100 W/cm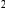) were sufficient to increase ROS concentration and to burst up the fluorescence signal inside the cells (Fig. 2c and 2d).

**Figure 3:**
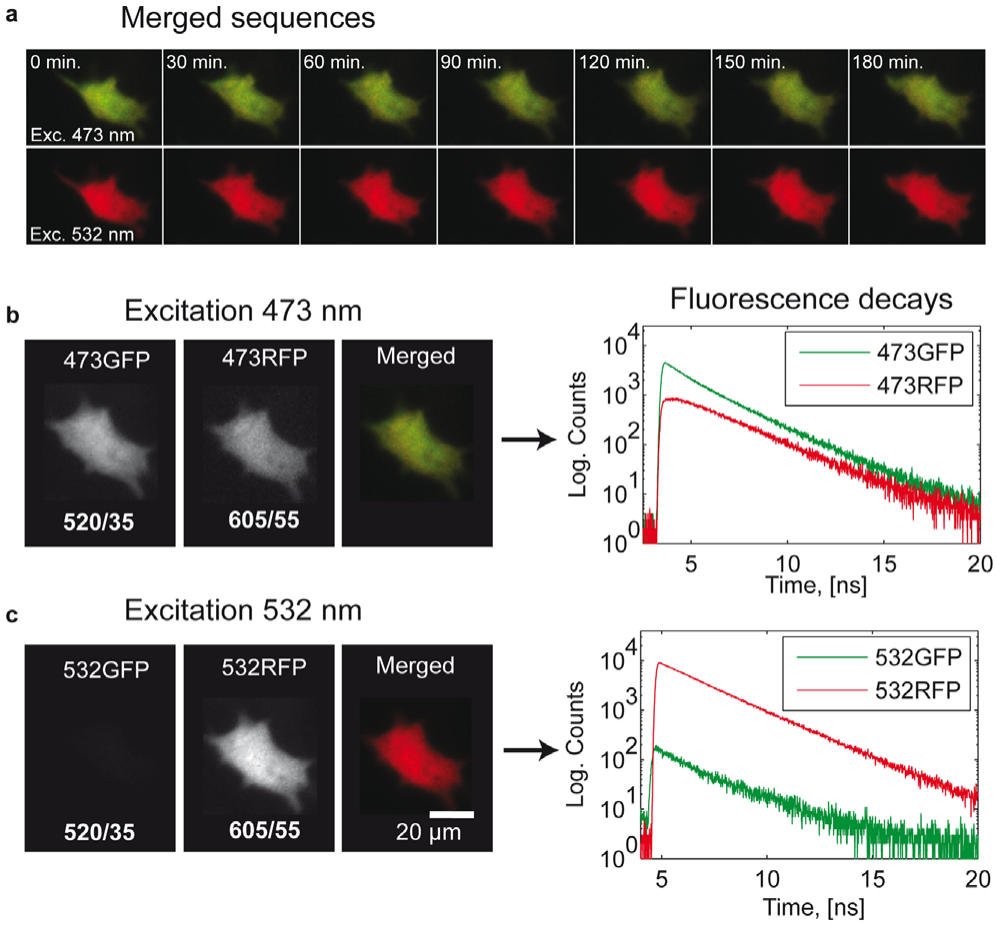
Long-term WFMP-FLIM experiment on HeLa cells. (a) 180 minutes of continuous WFMP-FLIM exposure of a living HeLa cell expressing a tagRFP-5AA-eGFP binary construct by pulsed interleaved excitation does not induce any detectable photobleaching. The two image streams are pseudo colored: eGFP (green) and tagRFP spectral detection channels (red). (b) Fluorescence decays in the 473GFP (green), 473RFP (red) and (c) 532GFP (green) and 532RFP (red) detection channels. The 473GFP/473RFP and the 532GFP/532RFP channels have been merged in pseudo-color images.

Multi-exponential lifetime analysis of the fluorescence decay of tagRFP-5AA-eGFP molecules after expression in HeLa cells clearly revealed that FRET occurred within the construct. Figure 3 depicts a summary of all the data simultaneously obtained from a single cell during one single experiment of 3 h exposure time (the complete experiment is shown in Video S1).

The fluorescence decay kinetics in the 473GFP and 473RFP FLIM channels (see Table 1 for a summary of the FLIM channels) are compared in Fig. 3b and 3c. The eGFP fluorescence measured in the 473GFP channel is visibly quenched and the signal measured in the 473RFP channel shows a slower rising edge of the fluorescence decay trace typical for FRET. Another evidence of FRET was obtained by analyzing the fluorescence decays of the construct in the tagRFP detection channel after PIE at 473 nm and 532 nm. Directly excited tagRFP (Fig. 3c, 532RFP) exhibited a shorter fluorescence decay compared to indirect excitation at 473 nm via eGFP mediated FRET (473RFP). Indeed, the radiative decay of tagRFP occurred after excitation transfer from the donor eGFP molecules with a finite time constant. The weak fluorescence signal, which was present in the 532GFP band, was attributed to the eGFP emission after excitation at 532 nm. As outlined in the *materials and methods* section, a 532 nm-notch filter was used to suppress the laser stray light in that channel. In fact, the laser excitation overlapped spectrally with the fluorescence signal of the eGFP molecules.

**Table 1:** Description of the four simultaneously acquired FLIM channels.

Channel name	Excitation wavelength	Detection wavelength	Applications
**473GFP**	473 nm (donor)	520/35 nm (donor)	Fluorescence signal of the donor molecules (i.e. natural lifetimes and the fast donor quenching due to FRET). May also show immature acceptor species excited at donor wavelength (e.g. DsRed)
**473RFP**	473 nm (donor)	605/55 nm (acceptor)	FRET mediated fluorescence signal of the acceptor molecules (i.e. rise of the fluorescence, bleed through of donor fluorescence and direct excitation of the acceptor molecules)
**532GFP**	532 nm (acceptor)	520/35 nm (donor)	Fluorescence signal of the donor molecules after excitation at wavelengths with a major acceptor absorption. May also contain the fluorescence of photo-converted molecules during the exposure time.
**532RFP**	532 nm (acceptor)	605/55 nm (acceptor)	Natural fluorescence signal of the acceptor molecules.

The experimental fluorescence decay data were subsequently analyzed according to the delta-function iterative re-convolution technique. For the current experiments, an Erythrosine B water solution with a mono-exponential decay of 86 ps was used as the reference fluorophore [22].

The parameters of the multi-exponential models were linked as already suggested by Laptenok et al. [23] and the results are presented in Table 2. The value of 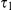 was linked between the 473GFP and 473RFP channels to improve the estimation accuracy. Remarkably, the pre-exponential factor of 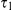 was present in the fluorescence emission of the tagRFP-5AA-eGFP construct in both 473GFP and 473RFP detection channels, respectively with a positive and negative value. This component of 0.44 ns was neither present in absence of the acceptor (HeLa cells expressing only eGFP, Table 3) nor after direct illumination of the acceptor at 532 nm indicating that it was clearly attributed to FRET.

**Table 2:** Result of the global analysis on single HeLa cells.

tagRFP-5AA-eGFP							
**Channel**	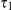 = 0.44 ns	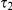 = 1.64 ns	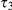 = 2.49 ns	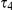 = 2.71 ns	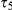 = 0.10 ns	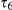 = 0.97 ns	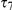 = 2.28 ns
	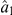 [%]	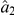 [%]	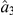 [%]	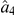 [%]	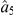 [%]	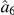 [%]	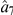 [%]
**473GFP**	42 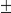 3	41 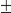 1	/	17 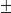 4	/	/	/
**473RFP**	−33 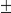 1	/	67 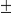 1	/	/	/	/
**532RFP**	/	/	/	/	25 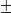 2	15 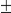 2	60 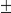 2

Cells expressing the tagRFP:5AA:eGFP construct.

**Table 3:** Result of the global analysis on single HeLa cells.

eGFP Alone			
**Channel**	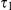 = 0.18 ns	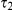 = 1.72 ns	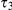 = 2.71 ns
	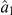 [%]	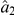 [%]	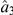 [%]
**473GFP**	17 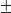 3	28 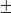 2	55 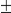 4

Cells expressing only eGFP, negative FRET control.

Result of the global analysis on single HeLa cells. In total, seven exponential components were resolved for cells expressing the tagRFP-5AA-eGFP construct: three for the 473GFP channel, two for the 473RFP and three for the 532RFP one. The normalized pre-exponential factors are shown. The errors were evaluated by confidential intervals at 68%. Standard deviations of the amplitudes were obtained from 7 cells expressing the tagRFP-5AA-eGFP construct and 11 cells expressing eGFP.

The observed eGFP decay kinetic was heterogeneous. However, several groups reported multi-exponential fluorescence decay kinetics of purified eGFP aqueous solutions (PBS buffer, pH 7.3). K. Suhling and coworkers [24] proposed a bi-exponential model (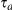 = 1.5 ns, 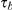 = 2.9 ns), which was attributed to two emitting species of the fluorophore. These lifetimes are similar to 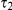 and 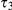 reported in Table 3. The fast component measured in cells expressing only eGFP (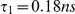) might be caused by the auto-fluorescence. In fact, untransfected HeLa cells showed a very complex decay kinetic of the fluorescence signal in the 473GFP channel (data not shown).

The apparent FRET efficiency 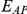, which is obtained assuming that all the donor molecules undergo FRET, and the real FRET efficiency (E) can be estimated 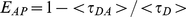
(2)

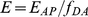
(3)where 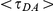 = 1.32 ns and 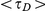 = 2.01 ns are the mean lifetimes (eq. 11) of the donor in presence and absence of the acceptor respectively and 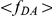 is the fraction of donor molecules, which undergo FRET (see below). Mean lifetimes (eq. 11) were used to account for the multi-exponential decay of the eGFP in spite of the average lifetimes (eq. 9). The obtained efficiencies were: 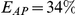 and 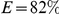. The spreading between these two values is due to a large fraction of eGFP molecules which are not involved in FRET (58% of the total). This value was correlated on the elapsed time between the transfection and the measurement of the cells (data not shown). When the auto-fluorescence contribution (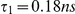) was removed and the pre-exponential factors were re-normalized, the mean lifetime of the eGFP became 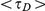 = 2.37 ns. Considering only the FRET active molecules present in cells expressing the eGFP-5AA-tagRFP construct (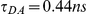), the real FRET efficiency was directly derived. 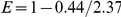 = 0.81 (81%), practically the same value obtained in the previous calculation. The average distance 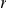 between the donor and acceptor molecules is given by 
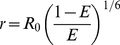
(4)where 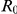 is the Förster radius, the donor-acceptor distance at which the real transfer efficiency is 50%. Assuming a random orientation of the donor and acceptor molecules (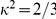) and a refractive index of 1.3, a Förster radius of 5.8 nm was elsewhere calculated [25]. The estimated average distance between the eGFP and tagRFP molecules in the measured construct was 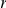 = 4.5 nm. Similar values were obtained by another binary FRET system composed of a eGFP and a mCherry molecule separated by a 6 amino acids linker [26]. In that case, an average FRET distance distribution peaked at 4.6 nm and a 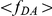 close to 50% were obtained.

**Figure 4:**
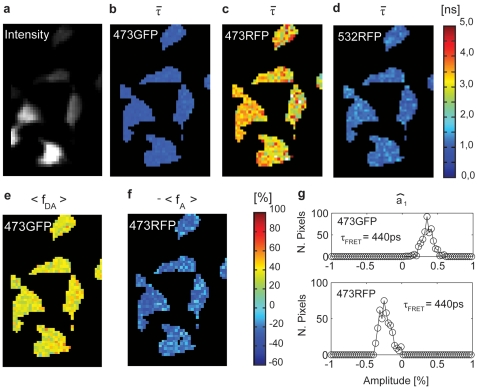
Spatial distribution of the average lifetimes. WFMP-FLIM image of tagRFP-5AA-eGFP transfected HeLa cells. (a) Intensity image. (b) Spatial distribution of the average lifetimes in the 473GFP, (c) 473RFP and (d) 532RFP channels. Spatial distribution of (e) 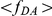 and (f) 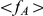. (g) Histogram of the parameters 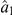 in the 473GFP and 473RFP channels. In the 473RFP channel the pre-exponential factor assumes negative values.

The pre-exponential factors of Table 2 were evaluated for each pixel of the set of the multi-parameter FLIM images. The pre-exponential factors provide stoichiometric information about the fluorophores' states e.g. percentage of the donor molecules undergoing FRET (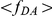) and the percentage of FRET-mediated excitation of the acceptor molecules (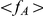). The parameters 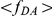 and 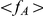 were calculated in each pixel (Fig. 4e and 4f). These physical quantities did not differ between nearby cells even if the expression levels were variable, suggesting that the tagRFP-5AA-eGFP construct had a homogeneous distribution within the sets of analyzed living HeLa cells. The same results were obtained in the calculation of the average lifetimes (Fig. 4b, 4c and 4d). The performed data analysis was successful even at very low numbers of photons per pixel. Fig. 4g shows the distribution of the pre-exponential factors 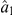 (associated to the FRET rate) in the 473GFP and 473RFP channels. Even less than 500 photons per pixel were sufficient to clearly discriminate negative (473RFP) and positive (473GFP) pre-exponential factors in the donor and acceptor channels.

### Mitochondria trafficking

**Figure 5:**
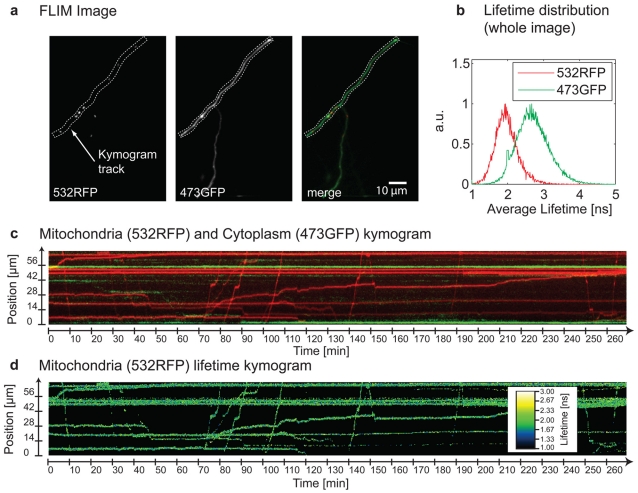
Two-color and lifetime kymogram. (a) Double transfected neuronal process: (red) tagRFP marked mitochondria and (green) eGFP in cell cytoplasm. (b) Distribution of the average lifetimes of eGFP and tagRFP molecules in the 473GFP and 532RFP FLIM channels. (c)The movement of the mitochondria and the cytoplasm flux are simultaneously measured and displayed in form of a kymogram. The sample was continuously measured for about 270 minutes. (d) Average lifetime kymogram of the tagRFP-labeled mitochondria. For each pixel of the kymogram, the average lifetime in the 532RFP channel was estimated. The average lifetime of the tagFRP-marked mitochondria were nearly constant during the measurement time and did not vary significantly.

WFMP-FLIM is capable to achieve the optical diffraction limit. To demonstrate the high spatial resolution of our setup for live cell imaging, we performed several tracking experiments on mitochondria-tagRFP transfected neurons. Neuronal processes of cells expressing moderately tagRFP-mito were continuously illuminated by PIE at 473 nm and 532 nm and the mitochondrial traffic was monitored for up to 10 h uninterruptedly (Video S2). We co-transfected the neurons with an eGFP vector, in some additional cases, to define the borders of the processes more accurately and to discriminate motor-protein driven mitochondrial transport from cytoplasmic diffusion. Three different categories of mitochondrial traffic (Fig. 5c) were observed in tagRFP-mito expressing neurons in agreement with previous observations [27]–[30]: a stationary population, an oscillatory population and (bi-)directionally moving population with an average speed of more than 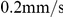. Transitions between these different states were accompanied by more or less distinct changes in mitochondrial morphology. Whereas moving mitochondria usually displayed an elongated shape of variable length, stationary mitochondria showed a more ovoid appearance. Transitions between resting, oscillating and moving phases were frequently found as well as fission and fusion [31] between moving and stationary mitochondria. The intensity kymogram in Fig. 5c demonstrates the complex dynamics of mitochondrial traffic consisting of fast movements, oscillatory periods and resting intervals. Particularly, the time traces of long-term stationary mitochondria verified an almost constant fluorescence emission without photo-bleaching over the whole recording period. eGFP and tagRFP-mito co-transfected neurons further revealed that mitochondria movements were mainly independent from cytoplasmic streaming (Fig. 5c). In addition to the intensity based kymograms of tagRFP and eGFP, the WFMP-FLIM method enables us to present, for the first time, a lifetime based kymogram of moving mitochondria (Fig. 5d). As both, intensity and lifetime, kymograms remained nearly constant they confirm that neither considerable photobleaching occurred during the whole exposure time nor photodynamic reactions influenced the average lifetime of tagRFP-marked mitochondria.

## Discussion

Wide-field FLIM by the Quadrant Anode detector is a valuable technique to measure the fluorescence decay of fluorophores at very high temporal resolution. The unique feature of the detector, i.e. the combination of a position sensitive photomultiplier and TCSPC, makes it a very sensitive tool for FLIM experiments in living cells [32]–[34].

The detection system has been extended to feature the simultaneous acquisition of multiple parameters of the fluorescence radiation: four simultaneous FLIM channels were obtained by combining Pulsed Interleaved Excitation (PIE) and multi-spectral detection.

Instead of PIE, an ALternating-Laser Excitation (ALEX) scheme could have been implemented to obtain a two-laser excitation of the sample [35]. In ALEX, a set of Electro-Optical Modulators (EOM) are inserted in the illumination path to switch sequentially the laser lines which illuminate the sample at frequencies greater than 1 kHz. The two techniques are both valid considering the experiments presented in this work. However, PIE was preferred because it does not induce any additional illumination losses due to the presence of the EOMs placed in front of the laser heads and the cross-talk between the excitation channels is practically zero. This last statement is valid only if the lifetime of the excited state of the fluorophores is not longer than 10–20 nanoseconds. Indeed, the fluorescence decay signal is completely vanished during the delay time between the interleaved pulses (60 ns in the current setup). Thus, the probability to detect a photon in the wrong channel is negligible.

The presented Wide-Field Multi-Parameter FLIM setup (WFMP-FLIM) provides several advantages compared to the available FLIM techniques concerning the long-term observation of living cells.

Laser Scanning Microscopy(LSM)-FLIM based systems expose the sample to higher peak intensities which might accelerate photobleaching or the photo-production of ROS species [36]. Conversely, FLIM systems based on Gated Optical Intensifiers (GOI) have a limited time resolution because of the small number of time channels available. For example, a FRET constant of few hundredths of picoseconds can not be directly measured in those systems. In order to compare GOI-based FLIM systems and the Quadrant Anode detector at very low-illumination intensities, the concept of Photon Economy (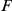) is useful [20]. 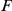 is an indicator of the efficiency of a FLIM setup in calculating average lifetimes. Given the number of photons collected, the lower the 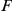-value, the better the estimation accuracy of the average lifetimes. The improvement of the accuracy is proportional to 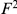. 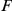-values of 1.5 and 1.23 were predicted by the theory for respectively 2 and 8 time-gates [1]. Conversely, the presented data analysis applied to the WFMP-FLIM data on Rhodamine 6G provided an equivalent 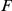-value of 0.65. The value of 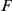 = 0.65 is only possible under the hypothesis of global analysis. A 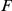-value greater than 1 is always obtained if the average lifetimes are independently calculated in each pixel of the same data set. Due to the 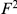 law, the WFMP-FLIM setup in combination with the presented data analysis is 5.5 and 3.6 times more accurate to estimate average lifetimes compared to the theoretical result of 2 and 8 time-gates GOI-based FLIM systems. Another advantage concerns the sensitivity at low photon detection-rates: the photons losses due to the dead time of the QA electronics are negligible in that regime. Most of the photons which interact with the photocathode are registered. In conventional GOI-based FLIM systems, only the photons which reach the intensifier when the gate is “on” are detected. The overall detection sensitivity is therefore decreased, particularly, when four or more time-gates are used. In conclusion, the 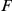-values achievable by the WFMP-FLIM setup are very useful when light-sensitive systems are measured. In fact, the method optimizes the accuracy of the parameter estimation at a given number of detected photons.

The performed experiments show that the advantages provided by WFMP-FLIM acquisition are feasible for long-term observation times on sensitive living systems. Exposure times of many hours were achieved both in HeLa and neuronal cells. The photobleaching rate and ROS production can be increased under the common illumination conditions required for FLIM imaging, depending on the observed fluorophores. An increased photobleaching-rate is a serious drawback for long-term experiments on living cells because it accelerates the fading of the fluorescent markers. An important example of the described behavior is the photobleaching-rate of the eGFP, the enhanced variant of the wild-type GFP from *Aequorea victoria*, as shown elsewhere [9]. The same non-linear bleaching behavior of the eGFP was independently verified in the current work (Fig. 2a). In parallel to photobleaching, the production of ROS may lead to premature cell death during short observation-time periods or alter the cell physiology due to the interaction with macro-molecules like proteins, lipids and nucleic acids. Like irreversible photobleaching, ROS production is fostered at high illumination intensities in the eGFP [37]. Therefore, eGFP was chosen as fluorescent marker for the long-term experiments on living cells, due to the above mentioned properties and its wide-spread use in many fields of the life-sciences, to test reliably the benefits of the WFMP-FLIM setup. In multiple transfection experiments, eGFP was measured in combination with tagRFP, a monomeric red-emitting fluorescence protein firstly derived from the sea anemone *Entacmaea quadricolor*. tagRFP was chosen due its superior properties concerning brightness, high quantum yield and pH-stability compared to other available monomeric fluorescent proteins emitting in the red-part of the visible spectrum. Furthermore, it shows a favorable Förster radius (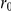 = 5.8 nm) for FRET applications if combined with the eGFP. The stability of tagRFP was sufficient during our long-term WFMP-FLIM experiments for exposure times up to 10 h although a more photostable mutant has been recently synthesized (tagRFP-T) [38].

Another advantage of the WFMP-FLIM setup is that the quantity of channels is only limited by the number of synchronized laser sources and image splitters inserted into the optical path. Therefore, the setup can be easily extended with additional channels to simultaneously measure the fluorescence anisotropy.

In co-localization experiments, only the 473GFP and 532RFP channels are employed corresponding to the dominant excitation and dominant emission of eGFP and tagRFP respectively. In FRET experiments, the fluorescence signal of the acceptor after donor excitation due to energy transfer is recorded in channel 473RFP. When FRET between donor and acceptor occurs, the following conditions must be simultaneously verified: a) the donor fluorescence shows a fast quenching component 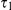 (473GFP channel) b) the acceptor fluorescence after direct excitation of the donor shows the same 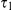 component but with a negative pre-exponential factor (473RFP channel) c) the 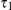 component is not present after direct excitation of the acceptor (532RFP channel) d) as long as there is no direct excitation by 532 nm of the donor (eGFP) or a photodynamic reaction (photoconversion/photoswitching) of the acceptor (tagRFP) to a blue shifted emission, the 532GFP channel should be always empty.

The simultaneous detection of the lifetime component with a negative amplitude (or rising component) in the 473RFP channel is very important. It increases the estimation accuracy of the FRET efficiency. In fact, the auto-fluorescence contribution to the signal, which spectrally overlaps with the eGFP fluorescence emission, might affect the values of the calculated parameters during the data analysis process. A bias in the obtained average donor-acceptor distance is then expected [23].

Considering that living cells are not static systems, only the simultaneous observation by multi-parameter FLIM can provide an accurate direct measurement of the FRET efficiency. The proposed WFMP-FLIM is conceptually equivalent to the intensity based FRET measurement via *Sensitized emission* as described by Gordon and coworkers [39]. However, the groundbreaking improvement of WFMP-FLIM is the supplementary information provided by the measurement of the fluorescence decay kinetics and the possibility to link model parameters, e.g. the lifetime of the donor or the Förster transfer rate, between different channels. It should be emphasized that the decay times (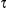) are not independently estimated from cuvette experiments, but they are directly measured from the observed cells.

The capability of WFMP-FLIM to follow subcellular structures was demonstrated by the tracking experiments on tagRFP labeled mitochondria. Long-term observations of these tiny organelles ranging in size from 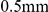 to 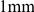 showed their ongoing bi-directional movements along neuronal processes independent of cytoplasmic streaming (Fig. 5c). Furthermore, the continuous acquisition of the mitochondria traffic revealed a more complex movement behavior made of abrupt phase changes (static, oscillating and moving) which cannot be resolved by time-lapse imaging with a CCD device over many hours. Intensity based as well as lifetime kymograms (Fig. 5d) of co-transfected neurons exhibited neither bleaching nor light-induced changes of their average lifetimes, indicating that the continuous low intensity laser illumination did not substantially disturb the cellular homeostasis. Since in the current (proof of principle) study, variations in average lifetimes of tagRFP labeled mitochondria could not be expected due to the dye's pH-stability [25], future investigations should address the question whether WFMP-FLIM measurements are suitable to correlate functional metabolic states of mitochondria with different movement patterns by using specific sensors for mitochondria's metabolism or membrane potential.

In summary our novel wide-field multi-parameter FLIM method, based on interleaved synchronized laser excitation of multiple fluorophores in combination with TCSPC by a space sensitive photomultiplier provides several advantages compared to current time-domain FLIM systems.

1. It allows long-term observation of living cells (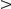
10 h) with continuous laser illumination at very low excitation intensities (
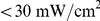
).
2. Simultaneous detection of donor and acceptor decay kinetics in spectrally separated channels leads to an enormous increase of information content during FRET experiments.

The WFMP-FLIM method might be used to disclose correlations between molecular interactions and metabolic changes since PIE is extensible to a third pulsed laser source for monitoring endogenous fluorescence of living cells (NAD(P)H or flavin cofactors as FMN and FAD). Moreover, incorporating a laser source for activation or switching of photoconvertible dyes into the PIE regime has the potential to combine WFMP-FLIM with super resolution imaging techniques like PALM, STORM and the dual Color Localization Microscopy (2CLM) [40]–[43], leading to fluorescence lifetime imaging nanoscopy.

## Materials and Methods

### Setup

**Figure 6:**
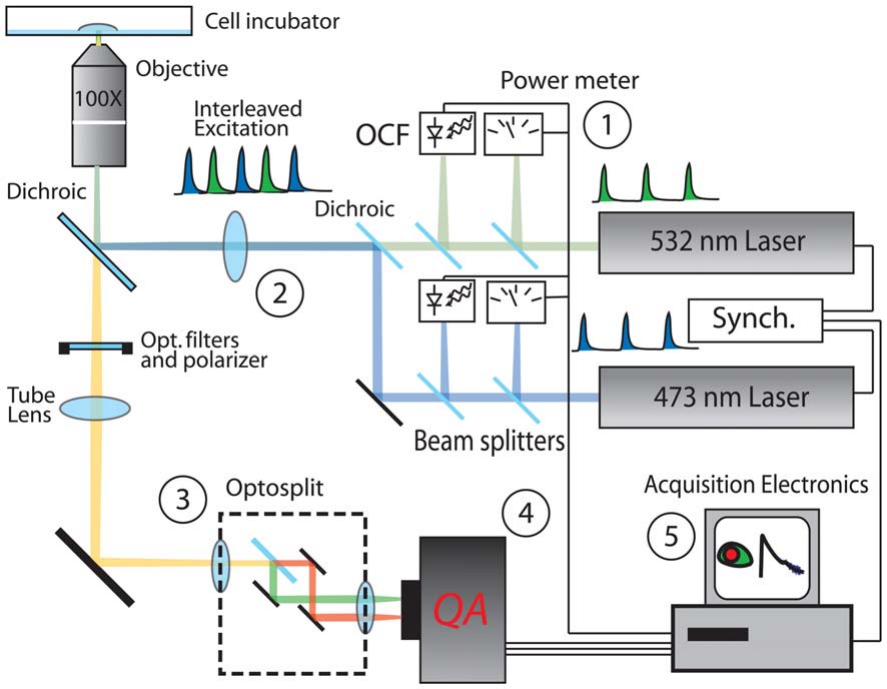
Scheme of the WFMP-FLIM experimental setup.

The WFMP-FLIM setup (Fig. 6) is based on a Nikon TI Eclipse wide-field fluorescence microscope equipped with autofocus (Nikon perfect focus) and 40X (N.A. 1.2) Plan Apo and 100X (N.A. 1.4) Plan Apo objectives (Nikon GmbH, Düsseldorf, Germany). (1) Two 8 MHz frequency-doubled Nd:Vanadate lasers tuned at 473 nm and 532 nm (HighQ Laser, Hohenems, Austria) were used as illumination sources. Both lasers generate pulses of comparable duration of about 10 ps at FWHM. The pulse repetition rate was synchronized by a phase-lock module (HighQ Laser, Hohenems, Austria). As result, two interleaved trains of pulses at an interval of about 60 ns were generated. This delay time is sufficient to separate the fluorescence emission of most organic dyes. Indeed, the fluorescence emission generated from one laser pulse is completely declined before the next excitation pulse arrives. A fraction of each laser beam was focused on two Optical Constant-Fraction discriminators (OCF-401, Becker & Hickl GmbH, Berlin, Germany) which delivered the synchronization pulses to the read out electronics. Average laser powers were measured every 10 seconds by power-meters (PD 300, Spiricon GmbH, Ahrensburg, Germany) to monitor variations in the illumination intensity during the experimental acquisition.

(2) The collimated interleaved trains of 473 nm and 532 nm laser pulses were merged and focused on the back focal plane of the objective to produce an uniform illuminated field of view. A dual band dichroic mirror (470-532-rdc, AHF, Tübingen, Germany) was used to discriminate between the excitation and the fluorescence signal. Several emission filters (532 nm Notch and 480LP AHF, Tübingen, Germany) removed the stray laser signals which were transmitted through the dichroic mirror. (3) The fluorescence signal collected via the objective was split into two spectral bands by a wide-field dual-imaging system (Optosplit II, Cairn Research Limited, Faversham, UK). A dichroic cube (565DXCR -Cairn Research Limited, Faversham, UK) and two band pass filters (520/35 and 605/55 AHF, Tübingen, Germany) were used to select the spectral channels. (4) The split images were projected on a Quadrant Anode (QA) photomultiplier (Europhoton GmbH, Berlin, Germany). Four spectral channels were simultaneously detected as summarized in Table 1.

(5) The original electronics of the QA detector has been extended to store a set of parameters for each collected photon in addition to the spatial coordinates. The synchronization signals from the OCFs were used to mark each detected photon with the wavelength (473 nm or 532 nm) of the last laser pulse which had illuminated the sample. A sequence of FLIM images with *instrumental response function* (*irf*) of 150 ps at a spatial resolution of about 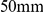 in the center of the detector active area were obtained after processing of the photon-stream. The time and the space bins were defined afterwards during post-processing of the stored data according to the requirements of the analysis.

The photobleaching experiments were performed illuminating the sample by a 150W Mercury lamp. Two Nikon filter sets, FITC (excitation 465 nm–495 nm, detection 515 nm–555 nm) and TRITC (excitation 530 nm–560 nm, detection 590 nm–650 nm), provided the required illumination and detection spectral channels.

The 100X Plan Apo objective was only used in the mitochondria tracking experiments.

### The model function of the fluorescence decay signal

The model of fluorescence decay signals acquired by the WFMP-FLIM setup is the numerical convolution between the *instrumental response function* (*irf*) of the TCSPC system and a decay model. The most commonly used model in the data analysis of FLIM decays is a linear combination of exponential functions or *multi-exponential model function*. The number of exponential functions (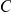) ranges between 1 and 4.

The pixels of the FLIM image are split in different subsets, and modeled according to the requirements of the global analysis i.e. in each subset the lifetime components (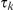) are common to all the pixels.

In each pixel subset, the fluorescence decay (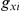) is modeled by the linear combination of normalized convolution integrals 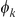. Additional measurements are required in order to correct for the *color effect* i.e. the *irf* shape is wavelength dependent in the adopted FLIM system. The common procedure is to adopt a special convolution procedure firstly introduced in the 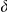 *-function iterative re-convolution method* [44]. Briefly, this method requires to measure the fluorescence decay signal of a reference solution (*ref*), whose decay kinetic has been previously characterized, instead of the *irf* by scattered laser light. The fluorescence signal of the *ref* dye is measured in all the detection channels as the probe. The *irf* of the reference dye and of the probe do not differ anymore because they are measured in the same spectral regions. In addition, the method requires that the reference compound decays following a mono-exponential kinetic. The non linear functions 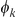 are expressed as the convolution between the reference decay signal *ref* and a modified model function (eq. 6).

Each pixel of the FLIM image is labeled by an unique index 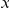 and the decay is calculated in each time channel, identified by the index 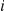.
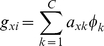
(5)

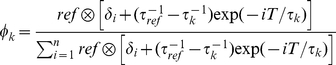
(6)

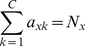
(7)where the coefficients of the linear combination 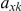 are named amplitudes, 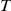 is the integration time of each channel in the fluorescence decay histogram and 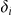 is a Dirac delta function. The lifetime component of the reference dye 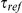 is assumed to be known or it is independently determined.

The sum of the amplitudes provide the number of the detected photons per pixel 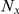. Generally the functions 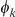 can also include a dynamic optical background or a constant function to model the detector dark counts. The parameters 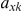 are then normalized to be independent from the number of photons in the pixel providing the normalized amplitudes 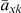


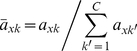
(8)where 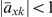. The parameters 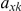 and 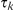 of the model functions are used to calculate the *average* lifetime (or intensity-averaged lifetime) of the fluorescence decay.
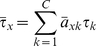
(9)


The average lifetime is also obtained by fitting a mono-exponential function to each fluorescence decay trace. However, in many applications especially the determination of the FRET efficiency from a donor with a multi-exponential decay kinetic, a different normalization of the amplitudes is required [6]. 
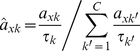
(10)

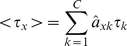
(11)where 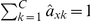 in each pixel. 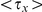 is commonly named *mean* lifetime (or amplitude-averaged lifetime) to be distinguish from the *average* lifetime of eq. 9. The parameters 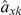 are termed normalized pre-exponential factors. The value of 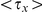 can not be obtained by a mono-exponential fit of the fluorescence decay trace of each pixel of the FLIM measurement. The only way to estimate 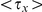 on a pixel basis is to fit multi-exponential decay models.

### FLIM data analysis algorithm

The estimation of the model parameters (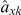 and 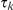) is performed in three steps:

#### Segmentation of the images

The pixels of the FLIM image are sorted in subsets by applying a segmentation algorithm as proposed by Pelet et al. [45]. The intensity of the fluorescence signal allows to select bright fluorescent cells on a darker background or to identify labeled regions within a transfected cell. Alternatively, manually defined regions of interest can be used. The pixels of each selected region are then assumed to be modeled by the same functions 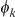 but with different parameters 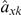 (*global analysis* hypothesis).

#### Estimation of the lifetime components

The fluorescence decay signals are summed in each pixel subset into a single decay trace whose total number of photons is very large. A multi-exponential decay model is fit to the sum of the data. The 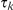 are obtained in each subset of pixels by 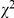 minimization (see for example [6]).

#### Estimation of the pre-exponential factors

Once the lifetimes are obtained, the pre-exponential factors are independently estimated in each pixel by minimization of the multinomial deviance 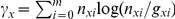 [46], where 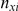 is the number of photons measured in the 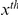 pixel and 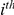 time-channel, 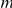 is the total number of time channels. For each 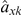, a FLIM map is generated to show its spatial distribution.

The analysis of the FLIM data was executed on a laptop computer (Acer Aspire 5930G with a Intel Centrino 2 P8600 (2.4 GHz) and 3 GB of RAM) in MATLAB (The MathWorks, Natick, MA), which provides different optimization functions. An intensity based discrimination was performed to identify different regions of the image and to remove the background pixels which contain no valuable information. A threshold value (between 30 to 50 photons per pixel) was applied during the segmentation procedure and the complementary regions were identified by a connected component labeling algorithm (*bwlabel*). The least-square problem was solved by using the *lsqnonlin* function based on the Levenberg-Marquardt algorithm. The minimization of the multinomial deviances 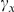 was performed by the *fmincon* function based on a reflective Newton method. During the minimization process, the pre-exponential factors have been constrained to obtain 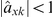.

### Ethics statement

Our experiments were done only on neuronal cell cultures obtained from rats which were sacrificed previously. According to the German animal welfare act (TschG, Art 4, Sect 3), these experiments are not regarded as animal experiments and, therefore, do not need an approval of an ethics committee.

### Cell cultures

The DNA of the plasmids was obtained by PCR encoding of tagRFP from pTagRFP-C vector (Evrogen, Moscow, Russia), adding a Kozak consensus sequence (accatggtg), the restriction site BamHI to the N-terminus and a short linker (SELRS) and the restriction site SacI at its C-terminus. DNA encoding eGFP with a short linker and the restriction site SacI at the N-terminus, the stop codon and the restriction site EcoRI at the C-terminus was obtained by PCR from pcDNA3-eGFP. Both fragments were digested with the indicated enzymes (Fermentas, St. Leon-Rot, Germany). In a second step, the mammalian expression vector pcDNA3 (Invitrogen GmbH, Karlsruhe, Germany) and the bacterial expression vector pRSET-B (Invitrogen, Karlsruhe, Germany) were digested with BamHI and EcoRI. Finally, the digested PCR fragments encoding tagRFP and eGFP were ligated into the pcDNA3 and pRSET-B vectors. All constructs were verified by nucleotide sequencing. Cells from a model cancer cell line (HeLa [47]) were cultured in Dulbecco's modified Eagle's medium (DMEM) without Phenol red supplemented with 10% fetal calf serum, 2 mM L-glutamine and 100 µ/ml penicillin at 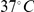 and 5% 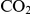 concentration. Cells plated on coverglass for 24 hours were transfected with Lipofectamine 2000 (Invitrogen) according to the manufacturer's protocol.

For long-term experiments the cells were measured in the described DMEM medium. For low background experiments, the culture medium was replaced 24–48 hours after transfection with Hanks Balanced Salt Solution supplemented with 1 g/L glucose and 10 mM Hepes (pH 7.4).

Photobleaching experiments were performed on paraformaldehyde-fixed cells (2% PFA in 0.1 M PBS (pH 7.4) for 10–15 minutes) to avoid any movements of the cells or metabolically related changes of the fluorophores (e.g. protein synthesis or degradation).

Preparation and culturing of the hippocampal neurons were performed according to the method of Banker et al. [48]. Cell cultures were derived from embryonic day 18/19 (E18/E19) rat embryos dissected from previously sacrificed rats of the institute's breeding facility. For preparation of high density cultures 60.000 to 80.000 cells and for medium density cultures 30.000 to 50.000 cells per petri dish were plated in DMEM including 10% Fetal calf serum, antibiotics (100 µ/ml penicillin, 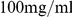 streptomycin) and 2 mM glutamine in 35 mm coded 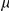-dish petri dishes (Ibidi, Munich, Germany) coated with Poly-D-Lysin. Cells were grown inside a Hereaus incubator with 5% 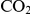 and 95% water-saturated air. 24 h after plating, the medium was completely exchanged with a Neurobasal medium without Phenol red, containing 2% B27, 1% antibiotics and 0.5 mM glutamine. Cells from different ages (DIV 5, 7, 9) were transfected with a tagRFP-mito construct or co-transfected with eGFP and tagRFP-mito using PolyFect Transfection Reagent (Qiagen GmbH, Hilden, Germany) or Lipofectamin 2000 (Invitrogen, Karlsruhe, Germany) and imaged 12 h to 36 h later. For long-term observation the cell cultures were maintained in a microscope incubator (Pecon, Erbach, Germany) during the whole experiment at controlled temperature (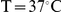) and 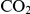 concentration and (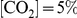).

## Supporting Information

Video S1 **Long-term WFMP-FLIM experiment on living HeLa cells.** The acquired FLIM channels are simultaneously shown. On the right side, fluorescence decay kinetics integrated over a region of interest.
(MOV)

Video S2 **Measurement of mitochondria trafficking by WFMP-FLIM.** Neuronal cell expressing tagRFP-mito marked mitochondria. The mitochondria trafficking was continuously monitored for 10 hours. Two kymograms were produced by selecting distinct processes. Anterograde and retrograde transport as well as oscillating and static phases are present. Mitochondria fissions and fusions are frequently visible.
(MOV)

## References

[1] Gadella TWJ (2008). FRET and FLIM Techniques, Volume 33..

[2] Bastiaens PI, Squire A (1999). Fluorescence lifetime imaging microscopy: spatial resolution of biochemical processes in the cell.. Trends Cell Biol.

[3] Jares-Erijman EA, Jovin TM (2006). Imaging molecular interactions in living cells by FRET microscopy.. Curr Opin Chem Biol.

[4] O'Connor D, Phillips D (1984). Time-Correlated Single Photon Counting..

[5] Becker W (2005). Advanced Time-Correlated Single Photon Counting Techniques..

[6] Lakowicz JR (2006). Principles of Fluorescence Spectroscopy..

[7] Borst JW, Hink MA, van Hoek A, Visser AJWG (2005). Effects of refractive index and viscosity on fluorescence and anisotropy decays of enhanced cyan and yellow fluorescent proteins.. J Fluoresc.

[8] Jares-Erijman EA, Jovin TM (2003). FRET imaging.. Nat Biotechnol.

[9] Bernas T, Zarebski M, Cook RR, Dobrucki JW, Cook PR (2004). Minimizing photobleaching during confocal microscopy of fluorescent probes bound to chromatin: role of anoxia and photon flux.. J Microsc.

[10] Hoebe RA, van Oven CH, Gadella TWJ, Dhonukshe PB, van Noorden CJF (2007). Controlled light-exposure microscopy reduces photobleaching and phototoxicity in fluorescence live-cell imaging.. Nat Biotech.

[11] de Vos WH, Hoebe RA, Joss GH, Haffmans W, Baatout S (2009). Controlled light exposure microscopy reveals dynamic telomere microterritories throughout the cell cycle.. Cytometry A.

[12] Prokazov Y, Turbin E, Vitali M, Herzog A, Michaelis B (2009). Reborn quadrant anode image sensor.. Nucl Instrum Methods Phys Res A.

[13] Müller BK, Zaychikov E, Bräuchle C, Lamb DC (2005). Pulsed interleaved excitation.. Biophys J.

[14] Millington M, Grindlay GJ, Altenbach K, Neely RK, Kolch W (2007). High-precision FLIM-FRET in fixed and living cells reveals heterogeneity in a simple CFP-YFP fusion protein.. Biophys Chem.

[15] Köllner M, Wolfrum J (1992). How many photons are necessary for fluorescence-lifetime measurements?. Chem Phys Lett.

[16] Verveer PJ, Squire A, Bastiaens PI (2000). Global analysis of fluorescence lifetime imaging microscopy data.. Biophys J.

[17] Laurence TA, Chromy BA (2010). Efficient maximum likelihood estimator fitting of histograms.. Nat Methods.

[18] Kay SM (1993). Fundamentals of Statistical Signal Processing: Estimation Theory..

[19] Bajzer Z, Therneau TM, Sharp JC, Prendergast FG (1991). Maximum likelihood method for the analysis of time-resolved fluorescence decay curves.. Eur Biophys J.

[20] Gerritsen HC, Asselbergs MAH, Agronskaia AV, van Sark WGJHM (2002). Fluorescence lifetime imaging in scanning microscopes: acquisition speed, photon economy and lifetime resolution.. J Microsc.

[21] Dixit R, Cyr R (2003). Cell damage and reactive oxygen species production induced by fluorescence microscopy: effect on mitosis and guidelines for non-invasive fluorescence microscopy.. Plant J.

[22] Boens N, Qin W, Basari N, Hofkens J, Ameloot M (2007). Fluorescence lifetime standards for time and frequency domain fluorescence spectroscopy.. Anal Chem.

[23] Laptenok SP, Borst JW, Mullen KM, van Stokkum IHM, Visser AJWG (2010). Global analysis of Förster resonance energy transfer in live cells measured by fluorescence lifetime imaging microscopy exploiting the rise time of acceptor fluorescence.. Phys Chem Chem Phys.

[24] Suhling K, Siegel J, Phillips D, French PMW, Lévêque-Fort S (2002). Imaging the environment of green fluorescent protein.. Biophys J.

[25] Merzlyak EM, Goedhart J, Shcherbo D, Bulina ME, Shcheglov AS (2007). Bright monomeric red fluorescent protein with an extended fluorescence lifetime.. Nat Methods.

[26] Visser AJWG, Laptenok SP, Visser NV, van Hoek A, Birch DJS (2010). Time-resolved FRET fluorescence spectroscopy of visible fluorescent protein pairs.. Eur Biophys J.

[27] Hollenbeck PJ, Saxton WM (2005). The axonal transport of mitochondria.. J Cell Sci.

[28] MacAskill AF, Kittler JT (2010). Control of mitochondrial transport and localization in neurons.. Trends Cell Biol.

[29] Chen S, Owens GC, Edelman DB (2008). Dopamine inhibits mitochondrial motility in hippocampal neurons.. PLoS ONE.

[30] Chen S, Owens GC, Crossin KL, Edelman DB (2007). Serotonin stimulates mitochondrial transport in hippocampal neurons.. Mol Cell Neurosci.

[31] Chan DC (2006). Mitochondrial fusion and fission in mammals.. Annu Rev Cell Dev Biol.

[32] Kemnitz K, Pfeifer L, Paul R, Coppey-Moisan M (1997). Novel detectors for fluorescence lifetime imaging on the picosecond time scale.. J Fluoresc.

[33] Jose M, Nair DK, Altrock WD, Dresbach T, Gundelfinger ED (2008). Investigating interactions mediated by the presynaptic protein Bassoon in living cells by Förster resonance energy transfer and fluorescence lifetime imaging microscopy.. Biophys J.

[34] Tramier M, Gautier I, Piolot T, Ravalet S, Kemnitz K (2002). Picosecond-hetero-FRET microscopy to probe protein-protein interactions in live cells.. Biophys J.

[35] Kapanidis AN, Lee NK, Laurence TA, Doose S, Margeat E (2004). Fluorescence-aided molecule sorting: analysis of structure and interactions by alternating-laser excitation of single molecules.. Proc Natl Acad Sci U S A.

[36] Petrášek Z, Eckert HJ, Kemnitz K (2009). Wide-field photon counting fluorescence lifetime imaging microscopy: application to photosynthesizing systems.. Photosynth Res.

[37] Greenbaum L, Rothmann C, Lavie R, Malik Z (2000). Green fluorescent protein photobleaching: a model for protein damage by endogenous and exogenous singlet oxygen.. Biol Chem.

[38] Shaner NC, Lin MZ, McKeown MR, Steinbach PA, Hazelwood KL (2008). Improving the photostability of bright monomeric orange and red fluorescent proteins.. Nat Methods.

[39] Gordon GW, Berry G, Liang XH, Levine B, Herman B (1998). Quantitative fluorescence resonance energy transfer measurements using fluorescence microscopy.. Biophys J.

[40] Schermelleh L, Heintzmann R, Leonhardt H (2010). A guide to super-resolution fluorescence microscopy.. J Cell Biol.

[41] Betzig E, Patterson GH, Sougrat R, Lindwasser OW, Olenych S (2006). Imaging intracellular fluorescent proteins at nanometer resolution.. Science.

[42] Rust MJ, Bates M, Zhuang X (2006). Sub-diffraction-limit imaging by stochastic optical reconstruction microscopy (STORM).. Nat Methods.

[43] Gunkel M, Erdel F, Rippe K, Lemmer P, Kaufmann R (2009). Dual color localization microscopy of cellular nanostructures.. Biotechnol J.

[44] Boens N, Tamai N, Yamazaki I, Yamazaki T (1990). Picosecond single photon timing measurements with a proximity type microchannel plate photomultiplier and global analysis with reference convolution.. Photochem Photobiol.

[45] Pelet S, Previte MJR, Laiho LH, So PTC (2004). A fast global fitting algorithm for fluorescence lifetime imaging microscopy based on image segmentation.. Biophys J.

[46] Maus M, Cotlet M, Hofkens J, Gensch T, de Schryver FC (2001). An experimental comparison of the maximum likelihood estimation and nonlinear least-squares fluorescence lifetime analysis of single molecules.. Anal Chem.

[47] Masters JR (2002). Hela cells 50 years on: the good, the bad and the ugly.. Nat Rev Cancer.

[48] Banker G, Goslin K (1998). Culturing Nerve Cells, Second Edition.. The MIT Press.

